# A systematic review of post-deployment injury-related mortality among military personnel deployed to conflict zones

**DOI:** 10.1186/1471-2458-9-231

**Published:** 2009-07-13

**Authors:** Joseph J Knapik, Roberto E Marin, Tyson L Grier, Bruce H Jones

**Affiliations:** 1 US Army Center for Health Promotion and Preventive Medicine, Aberdeen Proving Ground, MD, USA; 2 Occupational Medicine Department, Womack Army Medical Center, Fort Bragg, NC, USA

## Abstract

**Background:**

This paper reports on a systematic review of the literature on the post-conflict injury-related mortality of service members who deployed to conflict zones.

**Methods:**

Literature databases, reference lists of articles, agencies, investigators, and other sources were examined to find studies comparing injury-related mortality of military veterans who had served in conflict zones with that of contemporary veterans who had not served in conflict zones. Injury-related mortality was defined as a cause of death indicated by International Classification of Diseases E-codes E800 to E999 (external causes) or subgroupings within this range of codes.

**Results:**

Twenty studies met the review criteria; all involved veterans serving during either the Vietnam or Persian Gulf conflict. Meta-analysis indicated that, compared with non-conflict-zone veterans, injury-related mortality was elevated for veterans serving in Vietnam (summary mortality rate ratio (SMRR) = 1.26, 95% confidence interval (95%CI) = 1.08–1.46) during 9 to 18 years of follow-up. Similarly, injury-related mortality was elevated for veterans serving in the Persian Gulf War (SMRR = 1.26, 95%CI = 1.16–1.37) during 3 to 8 years of follow-up. Much of the excess mortality among conflict-zone veterans was associated with motor vehicle events. The excess mortality decreased over time. Hypotheses to account for the excess mortality in conflict-zone veterans included post-traumatic stress, coping behaviors such as substance abuse, ill-defined diseases and symptoms, lower survivability in injury events due to conflict-zone comorbidities, altered perceptions of risk, and/or selection processes leading to the deployment of individuals who were risk-takers.

**Conclusion:**

Further research on the etiology of the excess mortality in conflict-zone veterans is warranted to develop appropriate interventions.

## Background

There has been long-standing public concern about the health of service members following deployments to areas of military conflict. After World War II, the focus appears to have been on the well-being of repatriated prisoners of war [1-4]. In the Vietnam era, there was public alarm about Soldiers and Airmen exposed to the herbicide Agent Orange [5-7] and unease about post-service drug- and alcohol-related problems [8,9]. At the conclusion of the First Persian Gulf War, the vague and persistent complaints of service members, labeled "Gulf War syndrome" [10], lead to the development of a Gulf War registry and the expenditure of an estimated 1 billion dollars or more on evaluating Gulf War veteran health [11,12].

While the focuses of the many studies of post-conflict mortality varied and their findings were often equivocal [11,13-16], one relatively consistent finding did emerge. That finding was that the post-conflict injury-related mortality appeared to be higher among veterans who served in conflict zones compared with contemporary veterans who did not deploy to the conflict zones. In some studies, much of the excess mortality appeared to be associated with motor vehicle incidents.

The purpose of this paper is to systematically review the literature on the post-deployment injury-related mortality of service members who served in conflict zones. The paper also provides hypotheses as to why injury-related mortality may be higher among conflict zone veterans and reviews studies that have specifically tested some of these hypotheses.

## Methods

For this review, several retrieval databases were explored to find original, English-language studies on the post-conflict mortality of service members. The databases examined included PubMed (MEDLINE), the Cumulative Index to Nursing and Allied Health Literature, Academic Search Premier, Biomedical Reference Collection (Comprehensive), Ovid, and the Defense Technical Information Center. Keywords for the search included mortality, injury, motor vehicle accident, suicide, homicide, accident, war, and mishap with veteran, soldier, sailor, airmen, marine, service member, and military personnel. The reference lists of the articles obtained were also used to search for other studies and the files of some senior investigators were also reviewed. In some cases, investigators or agencies were contacted for more specific information.

One major consideration that surfaced during the review was selection of an appropriate control group. Comparing individuals serving in conflict zones with the general United States (US) population did not appear appropriate because of the "healthy worker" [17,18] or "healthy warrior" effect [19]. Generally, age-adjusted, all-cause mortality has been shown to be lower in veterans compared with civilians [1,2,7,18,20-29], presumably due to selection processes were by only healthier individuals are allowed and retained in the military services [18,30]. An alternative control group was non-conflict-zone service members serving at the same time as the conflict-zone service members. This approach was not without criticism since service members deployed to conflict zones may be healthier than those who do not deploy because of self-selection and medical screening processes [19]. To examine this potential problem, Kang and Bullman [31] examined the "healthy deployer" effect on mortality by comparing two groups of Reserve and National Guard veterans, one of which was deployed to nonconflict areas while the other was not deployed at all. The adjusted mortality rate ratio (deployed/not deployed) was 1.02 (95% confidence interval = 0.82–1.27) for all external causes (injuries), suggesting no mortality-related deployment selection bias.

With the above considerations in mind, studies were selected for review if they were original investigations in the English language and compared military service members who had served in conflict theaters with service members who had not served in the conflict theater but had been in military service at the same time as the conflict-zone service members. For selection, a study was required to include injury-related mortality in the analysis and to document the injury-related cause of death. Studies were excluded if they did not report injury-related mortality or if they included only service members who served in conflict zones (i.e., had no comparison group outside the conflict zone).

Injury-related mortality was defined as a cause of death indicated by International Classification of Diseases (ICD) E-codes E800 to E999 (external causes) or subgroupings within this range of codes. This code grouping includes causes of death not related to injury, notably accidental poisoning (E850–E869) and adverse effects from therapeutic drugs (E930–E949). In military studies that partitioned out accidental poisonings, the number of deaths ascribed to these was small, accounting for no more than 6% of all external cause deaths [9,29,32].

Meta-analysis was performed on selected articles that met the review criteria. The meta-analysis employed a general variance-based method involving confidence intervals [33]. This technique produced a summary mortality rate ratio (SMRR) and 95% confidence intervals (95%CI) for combined data from all selected studies.

## Results

A total of 5,144 unique studies were identified in the initial search. Two investigators examined the titles of these studies and looked at 156 abstracts from papers that appeared to meet the review criteria. After reading these abstracts, 49 articles were obtained for studies that appeared to involve injury-related mortality. These included 9 papers of veterans of the World II and the Korean conflict [1-4,18,34-37], 33 papers involving Vietnam veterans [5-7,9,20-27,29,30,38-56], and 7 papers involving veterans of the First Persian Gulf War [28,32,57-61].

The two reviewers concurred on 20 original investigations that fully met the review criteria. All of these investigations involved veterans of the Vietnam and the First Persian Gulf War eras. Studies involving veterans of World War II and the Korean conflict compared prisoners of war to other conflict-zone Soldiers [1-4,35], the conflict-zone status could not be determined [36,37], or the comparison group was the general population [3,4,18,34]. An additional eight reports provided useful methodological information on the 20 selected investigations [21,30,43-47,50].

Among the 20 investigations, 16 involved United States (US) service members [7,9,20,23-29,38-41,51,57], two involved United Kingdom (UK) service members [32,59], and 2 involved Australian service members [42,61]. Eight studies involved extended follow-ups of previously examined cohorts, a described in more detail below [24,26,29,32,40,50,51,57].

### Methodological issues in reviewed studies

Table 1 shows the methodologies used in the 20 selected veteran mortality studies. Methodological issues that arose during this review included the sensitivity of 1) veteran status, 2) conflict zone status, 3) vital status, and 4) cause of death coding. Sensitivity is defined as the proportion of cases correctly identified, divided by the total cases examined, expressed as a percent [62]. Since all the reviewed studies used databases (sometimes supplemented by personal interviews), sensitivity involves the proportion of agreement (%) between databases. Ideally, a criterion database (one known to be highly accurate) should have been identified and other databases compared with the criterion. Other methodological issues arising in this review included 1) use of different International Classification of Diseases (ICD) code books, 2) repeated follow-up studies on the same or similar cohorts, and 3) study design considerations. Additional studies were reviewed to assist in determining how these methodological issues affected the review.

**Table 1:** Methodological considerations in selected military veteran mortality studies

Study [Reference No]	Conflict	Source of Veteran Status	Source of Conflict Zone Status	Source of Vital Status	Cause of Death Coding	ICD Codebook Revision^c^	Methodological Quality Score
Proportionate Mortality Investigations							

Kogan & Clapp 1985 [7]	Vietnam	MA state bonus list^b^	MA state bonus list^b^	MA state mortality records	As listed in MA state mortality records and/or MA death certificates	ICD9	73

Lawrence et al. 1985 [20]	Vietnam	1. NY state vital records 2. DMDC	DMDC	1. VA-BLIRS 2. NY state vital records 3. Interviews	As listed in NY state vital records	ICD8	83

Anderson et al. 1986 [38]	Vietnam	1. WI state death certificate 2. VA-BIRLS 3. WI VA graves registration 4. Military service separation file (DD Form 214)	1. Military service separation file (DD Form 214) 2. WI VA graves registration	1. WI state mortality records 2. WI VA graves registration 3. VA-BIRLS	As listed on WI state death certificate	ICD7 (1960–67) ICD8 (1968–78) ICD9 (1979–85)	84

Breslin et al. 1988 [39]	Vietnam	VA-BIRLS	NPRC	VA-BIRLS	Nosologist used death certificate from VA files, state vital records (supplemented by SSA, IRS, & NDI for location of death), or DD casualty office	ICD8	80

Bullman et al. 1990 [40]	Vietnam	VA-BIRLS	NPRC	VA-BIRLS	Nosologist used death certificate from VA files or state vital offices	ICD8	79

Watanabe et al. 1991 [24]	Vietnam	VA-BIRLS	NPRC	VA-BIRLS	Nosologist used death certificate from VA files, federal archive records, or state vital offices	ICD8	79

Visintainer et al. 1995 [41]	Vietnam	MI state bonus list^b^	MI state bonus list^b^	MI death certificate database	As listed on MI death certificate	not stated	73

Watanabe & Kang 1996 [26]	Vietnam	VA-BIRLS	NPRC	VA-BIRLS	Nosologist used death certificate from VA files, federal records centers, or state vital offices	ICD8	80

Retrospective Cohort Investigations							

Fett et al. 1984 [42]	Vietnam	Australian national military service records	Australian national military service records	1. State & territory death registers 2. Electoral records 3. Arrival & departure records 4. Army records 5. Driver license records 6. Police/correction records 7. Welfare/credit records	Nosologist used death certificate supplemented with medical records, autopsy reports, legal reports, & coroner findings	ICD8	96

Boyle et al. 1987 [9]	Vietnam	NPRC	NPRC	1. VA-BIRLS 2. SSA 3. IRS 4. NDI 5. Army files 6. Interviews	Nosologist used death certificate	ICD8 ICD9	92

Thomas et al. 1991 [23]	Vietnam	1. VN group provided by respective services (see conflict zone status) 2. NVN group: Army-morning reports from 90 facilities; all other services-random sample of women never serving in VN	Provided by respective Services: 1. Army-morning reports from 91 VN facilities 2. Navy-muster rolls of the 4 VN naval facilities 3. Air Force-list of all women serving in VN 4. Marines-list of all women serving in VN	1. VA-BIRLS 3. SSA 4. IRS 5. NDI 6. Military Records	Nosologist used death certificate from VA files or state vital records offices	ICD8	88

Watanabe et al. 1995 [25]	Vietnam	Marine Corps file of all Marines on active duty	NPRC	VA-BIRLS	Nosologist used death certificate from VA files, federal records centers, or state vital offices	ICD8	84

Dalager & Kang 1997 [27]	Vietnam	1. DMDC (Chemical MOS) 2. Army Chemical School class rosters	Morning reports of chemical units in VN	1. VA-BIRLS 2. SSA 3. NDI	Nosologist used death certificate from VA files, federal records centers, or state vital offices	ICD9	85

Boehmer et al. 2004 [29]	Vietnam	NPRC	NPRC	1. VA-BIRLS 2. SSA 3. NDI Plus	1. NDI Plus 2. Nosologist used death certificate if cause of death not in NDI Plus	ICD9 (with NCHS modifications)	93

Cypel & Kang 2008 [51]	Vietnam	Rosters & personnel records from previous studies [23,27]	Rosters & personnel records from previous studies [23,27]	1. VA-BIRLS 2. SSA	1. NDI Plus 2. Nosologist used death certificate from VA files or federal records center	ICD9	82

Kang & Bullman 1996 [28]	Gulf	DMDC	DMDC	1. VA-BIRLS 2. SSA	Nosologist used death certificate from VA files or state vital offices	ICD9	89

Kang & Bullman 2001 [57]	Gulf	DMDC	DMDC	1. VA-BIRLS 2. SSA	Nosologist used death certificate from VA files, federal records centers or NDI	ICD9	87

MacFarlane et al. 2000 [32]	Gulf	UK Ministry of Defense	UK Ministry of Defense	Office for National Statistics, National Health Service Central Registers	1. Office for National Statistics performed coding 2. Defense Analytical Service Agency 3. Military sources in country of death	ICD9	83

MacFarlane et al. 2005 [59]	Gulf	UK Ministry of Defense	UK Ministry of Defense	Office for National Statistics, National Health Service Central Register	Office for National Statistics performed coding	ICD10	83

Sim et al. 2003 [61]	Gulf	Australian Defense Force	Australian Department of Veterans Affairs Nominal Roll for the Gulf War	Australian Institute of Health & Welfare NDI	As coded in Australian NDI	ICD9 (1990–1996) ICD10 (1997–2000)	86

^a^Abbreviations in table: VA-BIRLS = Veterans Administration-Beneficiary Identification and Record Locator Subsystem; NPRS = National Personnel Records System; DMDC = Defense Manpower Data Center; SSA = Social Security Administration; IRS = Internal Revenue Service; NDI = National Death Index; VA = Veterans Administration; NCHS = National Center for Health Statistics; VN = Vietnam veteran; NVA = not Vietnam veteran; MOS = military occupational specialty; DD = Department of Defense; MA = Massachusetts; MI = Michigan; WI = Wisconsin; UK = United Kingdom

^b^Bonus lists contain Vietnam-era service members who applied for and received money for service in the Vietnam era.

^c^ICD = International Classification of Diseases. The number after "ICD" indicates the codebook revision

#### Determination of veteran status and conflict-zone service

Veteran status was defined as serving or having served in the military. (Some veterans were still serving in the military at the time of the studies.) Conflict-zone status was defined as having served in a specific geographic area where hostile action occurred. Table 1 shows the sources used to identify veteran status and conflict zone status.

Among US sources, the Veterans Administration Beneficiary Identification and Record Locator Subsystem (VA-BIRLS) contains records of all compensation provided to US service members and their dependents (pensions, educational benefits, death benefits, home loans, life insurance, and other remunerations). The National Personnel Records Center (NPRC) is the repository for the medical and personnel records of discharged and deceased US service members after World War I. The Defense Manpower Data Center (DMDC) collects and archives data on US personnel and manpower and catalogues the history of military personnel.

A few studies provided some information on the sensitivity and completeness of veteran status from US sources. Veteran status concurred 95% to 98% when death certificates were compared with information obtained from personal interview with heads of households; however, veteran status was often missing on death certificates [20,63]. Deceased veterans identified first through NPRC records were subsequently found in VA-BIRLS records 82% to 93% of the time [64]. Veterans identified first though VA-BIRLS records were subsequently identified in NPRC records 98% to 99% of the time [24,26,39]. Identification of veterans in the NPRC appears to be much more complete than in the VA-BIRLS and thus NPRC records are likely the criterion source for US veteran status.

Bonus lists were the source of veteran and conflict-zone status in two studies [7,41]. These lists included individuals eligible for state monetary benefits for service during the Vietnam conflict. These bonuses had certain restrictions and verification requirements, as well as requiring active application on the part of the veteran. In addition, there was differential pay to those who served in Vietnam versus those who did not serve in Vietnam. It is possible that not all veterans were aware of or applied for the bonuses, that some subgroups (e.g., lower educational status, higher socioeconomic status) may have been underrepresented, and that the differential pay may have provided differential application incentives for Vietnam verses non-Vietnam veterans.

Military personnel records at the NPRC [46], records at the DMDC, and records at the UK Ministry of Defense contained information on the location of service for each service member, but no comparison studies were found. In an Australian study [42], Vietnam service status from a random sample of 2,000 Vietnam-era conscripts from the Melbourne Regional Computing Center was compared with conflict-zone status from the Central Army Records Office. Presence in Vietnam concurred in over 99% of cases from these two sources [42].

#### Vital status determination

Table 1 shows the sources for ascertainment of vital status in the veteran mortality studies. In the US, the National Death Index (NDI) is considered the criterion source for mortality determination since studies using independent samples of known mortality find that 97% to 98% of deaths are correctly accounted for in the NDI [29,65-71]. However, the NDI only contains mortality information beginning in 1979 [66,68,69].

Studies that examined the VA-BIRLS for vital status found that 70% to 95% of cases were in agreement with the NDI or known mortality from other sources [25,29,64,67,69,72-75]; some comparisons exceeded 90% concurrence [25,67,73,75]. The VA-BIRLS data were more accurate in reporting deaths of veterans serving in Vietnam compared with those not serving in Vietnam; this bias underestimates the deaths of non-Vietnam veteran [64,75]. Vital status obtained from the Social Security Administration (SSA) mortality files exhibited sensitivity similar to that of the VA-BIRLS: studies comparing vital status in the SSA to mortality from known sources found that 73% to 95% of cases concurred [29,69,72,75,76], with most studies exceeding 88% agreement [29,72,76]. Mortality data from the Internal Revenue Service (IRS) concurred with one known mortality source in only 43% of cases when the mortality search was limited to the 5 years for which the IRS retained their records [69].

Most retrospective cohort studies involving US veterans [9,23,27-29,57] determined vital status from multiple sources, and this has been shown to increase the sensitivity of ascertainment. For example, in Vietnam studies, if both VA-BIRLS and SSA data were used, the sensitivity of vital status detection increased to 96% or higher [29,69,72,75]. One Gulf War mortality study using vital status from combined VA-BIRLS and SSA sources found only 89% agreement with the NDI, but missing mortality cases were evenly distributed between the Gulf War and non-Gulf War cohorts thus reducing bias [28]. One cohort study [25] used only VA-BIRL vital status data, but when a random sample of 2,000 cases in this study was compared with the NDI, 95% of vital statuses were concordant. Half of the Vietnam proportionate mortality studies (most involving serial additions to the same initial cohort) used only VA-BIRLS for vital status determination [24,26,39,40].

In the Australian Vietnam study [42], vital status from the Australian Department of Veterans Affairs was compared with that obtained from Australian death registers. There was agreement in 95% of the cases from these sources.

In summary, vital status in many Vietnam proportionate mortality investigations using only VA-BIRLS [24,26,39,40] were questionable because of the lower sensitivity of this single source. Complicating this was bias introduced by the greater sensitivity in detecting Vietnam veteran deaths compared with non-Vietnam veterans' deaths [64,75], which would increase the likelihood of finding higher mortality in the Vietnam veterans. All but one of the retrospective cohort investigations involving US service members used multiple vital status sources, presumably improving vital status sensitivity to 96% or higher. The one retrospective cohort study using only VA-BIRLS for vital status found that 95% of a random sample of their cohort that were listed as deceased in VA-BIRLS were also listed as deceased in the NDI [25]. The Australian studies used multiple sources; a single Australian Army source had 95% concurrence with the national vital status source. No studies on vital status sensitivity in UK sources were found.

#### Cause of death determination

Table 1 shows information on cause of death coding in the veteran mortality studies. State death certificates are the major source of this information in the US. Kogan and Clapp [7] reported a 99% agreement between a Massachusetts mortality database and death certificates. In the Vietnam Experience Study [77], a nosologist's coding from death certificates (interrater reliability = 98% agreement) were compared with ratings from a medical panel (two physicians and one nurse). In addition to death certificates, the medical panel acquired information from law enforcement, hospitals, medical examiners, and other sources and used these data to make an independent determination of cause of death. Overall agreement between the death certificates and the medical panel on three-digit codes from ICD Revision 9 (ICD-9) was 97% for motor vehicle events, 90% for suicides, and 96% for homicides. Another study [66] compared cause of death codes from three nosologists with those coded in the NDI and found 96% agreement for three-digit ICD-9 codes for all-cause mortality.

In the Australian Vietnam study, 399 injury (external cause) deaths were reviewed by both the Australian Bureau of Statistics (personnel who normally code death certificates) and a panel of physicians with "experience as medical referees of the Crematorium Society of Australia" [42]. The two groups agreed on ICD-9 cause of death codes in 378 cases (95%). For motor vehicle events, suicides, and homicides, agreement was 98%, 98%, and 90%, respectively (there were only 10 homicides among the reviewed deaths).

In summary, agreement exceeds 95% when cause of death on death certificates (US and Australian sources) were compared with causes determined by various authorities for external causes and motor vehicle events. Agreement on suicide deaths in US sources may be less accurate, but agreement is still 90%. No studies on cause of death sensitivity in UK sources were found.

#### ICD codebooks and codes used in Vietnam studies

The penultimate column in Table 1 shows the edition of ICD codebooks used to determine injury-related cause of death in various studies. For most Vietnam studies, causes of death were coded using the ICD-8 [20,21,23-26,39,40,42] or ICD-9 codebook [7,9,27,29], but one study used ICD-7 to ICD-9 depending on the year of death [38], and one study did not specify the codebook used [41].

For all injury-related mortality, most Vietnam studies used the full range of "external cause" codes, E800 to E999 [7,9,24-26,29,38,40], although two studies used E800 to E998 [23,39], and one E800–E989 [42]. Codes E990 to E999 are injuries resulting from operations of war. Two studies did not provide the exact codes used [27,41].

For motor vehicle events, most Vietnam studies used codes E810 to E827 [23,24,26,38-40], but other studies used E810 to E825 [7,20,29], E810 to E825 plus E929.0 [9], E810 to E835 [25], and E810 to 824 [42]. Code E826 is mortality associated with bicycles (ICD-8 and ICD-9); code E827 is mortality as a pedestrian on a road in ICD-8 only. E830 to E835 includes watercraft transport-related mortality. E929.0 is late effects of motor vehicle injury in the ICD-9 codebook.

For suicides, almost all Vietnam studies used codes E950 to E959 [9,20,23-26,29,38-40,42]. One study used codes E950 to E958 [7]. Code E959 is the late effects of self-inflected injury.

For homicides, Vietnam studies used codes E960 to E969 [7,9,20,29,39] or E960 to E978 [24,26,40]. E970 to E978 includes mortality due to legal intervention by firearms, explosives, gas, blunt objects, other and unspecified means, as well as legal execution.

#### ICD-9 codebooks and codes used in Gulf War studies

Table 1 shows that most Gulf War studies used the ICD-9 codebook [28,32,57,61]. One study reported both ICD-9 and ICD-10 codes [61], while another used the ICD-10 codebook [59]. Studies employing the ICD-9 codes [28,32,57,61] used E800 to E999 for all external causes, E810 to E825 for motor vehicle accidents, E950 to E959 for suicide, and E960 to E969 for homicide. The study using the ICD-10 codebook [59] used all V codes (presumably V01–V99) for "transport accidents" and X60–X64 and Y10–Y34 for "intentional self harm."

#### Repeated examinations of the same or similar cohorts

The same or similar cohorts have been used in five groups of studies. The first group of studies involved a succession of four proportionate mortality investigations that used the same initial sample [39] and added deaths and/or subjects over time [24,26,40] for a total average 24-year follow-up in the latest report [26]. One of these studies used a subsample of Army veterans stationed in a specific geographic region in Vietnam [40]. Two studies involved the Vietnam Experience Study cohort of single-term enlisted soldiers [9,29]. One study combined female Vietnam veteran data from two previous investigations [23,50] for a total follow-up time of 32 to 40 years [51]. Almost the entire US military population serving during the Gulf War was initially examined at three years post-conflict [28] and then again at seven years post-conflict [57]. A similar cohort of UK Gulf War veterans was studied after average follow-up times of 8 and 13 years [32,59].

#### Study design

Studies were of two general designs: proportionate mortality and retrospective cohort investigations. Proportionate mortality (PM) studies considered only deaths. These studies calculated veteran deaths due to specific causes as a proportion of all veteran deaths (PM = ∑ cause-specific deaths/∑deaths from all causes). Mortality among veterans serving in conflict zones (PM_c_) and those not serving in conflict zones (PM_nc_) were compared (PM_c_/PM_nc_). An excess of deaths in one group may reflect an actual excess, but it may also reflect fewer deaths in other mortality categories.

Retrospective cohort studies considered both veterans who were deceased and those still alive. The mortality rate (MR) is calculated as MR = ∑ cause-specific deaths/∑alive and dead veterans. These studies identified groups of conflict-zone veterans (MR_c_) and non-conflict-zone veterans (MR_nc_) and calculated a mortality rate ratio comparing the two groups (MC_c_/MC_nc_). Retrospective cohort studies have the advantage of providing the cause-specific relative mortality rate in the two groups [78].

### Methodological quality rating

A rating system was developed to evaluate the methodological quality of the 20 reviewed investigations. The criteria for the ratings are shown in Table 2. Ratings were based on the methodological considerations described above, conversations with individuals knowledgeable on the various data sources, and examination of websites describing the data sources. Where objective data were available that compared a criterion database to another database, a score of 1 point was given for every 5% of agreement. Thus, 80% concurrence between a criterion data source and a rated data source would score 16 points for the rated source; 90% concurrence would score 18 points. Where objective data were not available, a judgment was made on the sensitivity of the database based on descriptions in articles, descriptions on websites, and conversations with individuals knowledgeable about the database. Additional points were given if additional data sources were examined for veteran status or vital status, since this would increase sensitivity and completeness of follow-up. Additional points were also given if the article provided independent confirmation of the sources of veteran status or vital status. In this rating scheme, the range of scores was 70 to 100 points. The last column of Table 1 shows that the methodological quality scores for the 20 reviewed studies ranged from 73 [7] to 96 points [42]. The retrospective cohort studies scored higher than the proportionate mortality studies.

**Table 2:** Methodological quality scoring system

Methodological Consideration	Sources – points	Number of Sources Examined – Points	Independent Confirmation of Sources – Points	Total Minimum Points Possible	Total Maximum Points Possible
Veteran Status	(Maximum Points = 20) US state bonus lists – 14 US state vital records – 18 US DMDC – 18 US VA-BIRLS – 18 US Graves Registration – 19 US Military Records (DD214) – 19 US NPRC – 20 US Special Lists – 16 Australian Service Records – 19 UK Ministry of Defense Records – 18	1 source – 0 2 sources – 1 3 sources – 2	Independent confirmation – 1	14	23

Conflict-zone Status	(Maximum Points = 20) US state bonus lists – 17 US DMDC – 19 US Military Records (DD214) – 18 US NPRC – 19 US VA-BIRLS – 18 US special lists – 16 Australian Service Records – 20 UK Ministry of Defense Records – 18			17	20

Vital Status	(Maximum Points = 20) US state vital records – 20 US VA-BIRLS – 17 US SSA – 18 US VA-BIRLS & SSA – 19 US graves registration -19 US IRS^b ^– 10 US NDI – 20 US Military Records – 19 UK Office of National Statistics – 20 UK Defense Analytic Service Agency – 19 Australian state and territorial registers – 20 Australian electoral records – 18 Australian death registers – 19 Australian immigration data – 14 Australian Army records – 19 Australian driver's license data – 17 Australian NDI – 19	1 source – 0 2 sources – 1 3 sources – 2 4 sources – 3 5 sources – 4 6 sources – 5	Independent confirmation – 2	17	27

Cause of Death Coding	(Maximum Points = 20) US state vital records – 17 US NDI Plus – 17 Australian NDI – 17 UK Office for National Statistics – 17 Nosologist using death certificate – 18 Nosologist using death certificate plus other sources – 20			17	20

Study Design	(Maximum Points = 10) Proportionate Mortality – 5 Retrospective Cohort – 10			5	10

Total Points	90	7	3	70	100

^a^Abbreviations in table: VA-BIRLS = Veterans Administration-Beneficiary Identification and Record Locator Subsystem; NPRC = National Personnel Records Center; DMDC = Defense Manpower Data Center; SSA = Social Security Administration; IRS = Internal Revenue Service; NDI = National Death Index; DD = Department of Defense; UK = United Kingdom, US = United States

^b^No study used IRS records alone to determine vital status

### Injury-related mortality among Vietnam veterans

Fifteen studies examined post-conflict, injury-related mortality of Vietnam veterans. Most studies involved discharged service members [7,9,20,21,25,27,29,38,41,42], but some included a few individuals who were likely still in service during the survey period [23,24,26,39,40].

The injury-related results of the Vietnam-era investigations are summarized in Table 3. These studies involved US and Australian service members. The proportionate mortality studies generally indicated that that the proportion of injury-related deaths (i.e., all external causes) was higher in veterans who served in Vietnam compared to those who did not. The studies that did not show a higher proportion of deaths in the Vietnam veterans involved more select groups of service members and/or smaller samples [38,39,41]. With one exception [20], all studies examining motor vehicle-related mortality showed a higher proportion of deaths among veterans who served in Vietnam. A few studies [20,26,41] showed a higher proportion of suicide-related mortality rates among veterans with Vietnam service, but most [7,24,26,38-40] showed little difference between veterans who served in Vietnam and those who did not. Most studies examining homicide-related mortality [20,24,26,38,40,41] suggested a slight excess among veterans with service in Vietnam.

**Table 3:** Studies examining injury-related post-Vietnam service mortality

	Study Characteristics				Mortality Rate Ratios Vietnam Veterans/Non-Vietnam Veterans (95% Confidence Intervals Where Available)			
	
Study [Reference No]	Follow-Up Period (years)^a^	Sample^b^	Sample Size^c^	Measure^d^	All External Causes (Injury)	Motor Vehicle Accidents	Suicide	Homicide
Proportionate Mortality Investigations								

Kogan & Clapp 1985 [7]	M = 10–25	US White ♂ Veterans from MA State	VN = 840 NVN = 2,515	PMR	1.08	1.10	0.93	0.80

Lawrence 1985 [20]	M = 7–10	US ♂ Veterans from NY State	VN = 555 NVN = 941	APMR^e^	no data in article	0.86 (0.66–1.11)	1.24 (0.88–1.75)	1.59 (0.86–2.94)

Anderson et al. 1986 [38]	M = 3–14	US White ♂ Veterans from WI State	VN = 922 NVN = 1,569	PMR	0.99 (0.96–1.03)	1.03 (0.95–1.11)	0.98 (0.84–1.15)	no data in article

Breslin et al. 1988 [39]	M = 8–17	US Army ♂ Veterans	VN = 19,708 NVN = 22,904	PMR	1.03 (1.02–1.04)	1.05 (1.01–1.09)	0.93 (0.88–0.98)	1.01 (0.73–1.40)
				
		US Marine ♂ Veterans	VN = 4,527 NVN = 3,781		1.00 (0.95–1.05)^f^	1.07 (0.97–1.18)	0.93 (0.86–1.01)	0.98 (0.89–1.08)

Bullman et al. 1990 [40]	M = 11–19	US Army Veterans	VN = 6,668 NVN = 27,917	PMR	1.06 (1.03–1.09)	1.08 (1.02–1.14)	0.97 (0.90–1.04)	1.07 (0.99–1.16)

Watanabe et al. 1991 [24]	M = 10–20	US Army ♂ Veterans	VN = 24,145 NVN = 27,917	PMR	1.03 (1.01–1.05)	1.03 (0.99–1.07)^f^	0.96 (0.91–1.01)^f^	1.02 (0.98–1.10)^f^
				
		US Marine ♂ Veterans	VN = 5,501 NVN = 4,505		1.02 (0.99–1.05)^f^	1.02 (0.95–1.12)^f^	0.99 (0.89–1.10)^f^	1.04 (0.93–1.17)^f^

Visintainer et al. 1995 [41]	M = 18–24	US ♂ Veterans from MI State	VN = 3,364 NVN = 5,229	PMR	0.95 (0.89–1.02)	no data in article	1.03 (0.93–1.14)	1.03 (0.93–1.14)

Watanabe & Kang 1996 [26]	M = 17–23	US Army ♂ Veterans	VN = 27,596 NVN = 31,757	PMR	1.04	1.03	0.97	1.05 (1.01–1.09)
				
		US Marine ♂ Veterans	VN = 6,237 NVN = 5,040		1.02	1.02	1.01	1.01

Retrospective Cohort Investigations								

Fett et al. 1984 [42]	M = 9–16	Australian ♂ Army Conscripts	VN = 19,205 NVN = 26,957	MRR	1.3 (1.0–1.3)	1.2 (0.9–1.5)	1.5 (0.9–2.3)	no data in article

Boyle et al. 1987 [9]	M = 12–18 A = 14	US Army ♂ Junior Enlisted Veterans	VN = 9,324 NVN = 8,989	MRR	1.25 (1.00–1.55)	1.48 (1.04–2.09)	0.98 (0.58–1.65)	0.99 (0.57–1.71)

Thomas et al. 1991 [23]	M = 15–23 A = 17	US ♀ Service Members	VN = 4,582 NVN = 5,324	AMRR^g^	1.33 (0.80–2.23)	3.19 (1.03–9.86)	0.96 (0.39–2.39)	no data in article

Watanabe et al. 1995 [25]	M = 18–24 A = 22	US Army Marines	VN = 10,716 NVN = 9,346	MRR	1.20 (0.99–1.45)	1.04 (0.76–1.43)	1.15 (0.75–1.76)	no data in article

Dalager & Kang 1997 [27]	M = 18–26 A = 20	US Army Chemical Corps Personnel	VN = 2,872 NVN = 2,737	AMRR^h^	0.83 (0.57–1.22)	no data in article	no data in article	no data in article

Boehmer et al. 2004 [29]	M = 29–35 A = 30	US Army ♂ Junior Enlisted Veterans	VN = 9,324 NVN = 8,989	MRR	1.19 (1.01–1.39)	1.24 (0.94–1.64)^i^	1.03 (0.74–1.44)^i^	0.90 (0.60–1.36)^i^

Cypel & Kang 2008 [51]	M = 32–40	US ♀ Service Members	VN = 4,586 NVN = 5,325	AMRR^j^	1.34 (0.91–1.96)	2.60 (1.22–5.55)	0.90 (0.44–1.85)	no data in article

^a^M = Maximal follow-up period (the first number is the year the last person entered the study to the end of the survey period; the second number is the year the first person entered the study to the end of the survey period). A = Average follow-up time (if reported).

^b^US = United States, MA = Massachusetts, MI = Michigan, NY = New York, WI = Wisconsin

^c^VN = Vietnam-era veterans serving in Vietnam; NVN = Vietnam-era veterans not serving in Vietnam

^d^PMR = proportionate mortality ratio; APMR = adjusted proportionate mortality ratio; MRR = mortality rate ratio; AMRR = adjusted mortality rate ratio

^e^Adjusted for age, race, and education

^f^Approximate confidence interval calculated from data in study

^g^Adjusted for age, race, rank, military occupational specialty, and duration of military service

^h^Adjusted for age, race, rank, and duration of military service

^i^Calculated from data in article

^j^Adjusted for rank, nursing status, duration of military service, age at entry, and race

The retrospective cohort investigations showed results similar to that of the proportionate mortality investigations. Most retrospective cohort investigations demonstrated that injury-related mortality was elevated in veterans who served in Vietnam compared with those that did not. The one study that did not show an excess of injury-related mortality for veterans serving in Vietnam involved a small group of Soldiers serving in chemical specialties [27]. All studies examining motor vehicle-related mortality showed higher mortality rates among veterans who served in Vietnam. Some investigations [25,29,42] showed higher suicide-related mortality rates among veterans with Vietnam service, but others [9,23,51] did not. The two investigations examining homicide-related mortality involved the same cohort of veterans examined at two separate times; the results suggested that initially (12–18 years of follow-up) there was little difference between veterans serving in Vietnam and those not [9] but later (32–40 years of follow-up), those serving in-country had lower homicide-related mortality.

A small portion of the excess external-cause mortality appears to be due to drug-related events. Two studies [9,29] involving the same cohort of US Army Soldiers showed an excess of deaths from accidental poisoning (ICD-9 E-codes E850–E869) over an average 30-year follow-up period (MRR(Vietnam veterans/non-Vietnam veterans) = 2.26, 95%CI = 1.12–4.57). When all-cause mortality was categorized to indicate all drug-related events (including those with E-codes outside the E800–E999 range), the Vietnam veterans experienced an excess of deaths for drug-related reasons over the 30-year follow-up (MRR (Vietnam veterans/non-Vietnam veterans) = 1.70, 95%CI = 1.01–2.86). However, accidental poisonings and drug-related events accounted for only 6% and 2%, respectively, of all external cause deaths.

Interestingly, in the studies of Australian Vietnam service members [21,42,43], adjustment for service corps (infantry, engineer, armor/artillery, minor field presence, no field presence) considerably reduced the mortality rate ratios. This was primarily because most of the excess mortality was limited to the engineers. In Vietnam, Australian engineers functioned in laying, detection, and disposal of mines, tunnel clearance, demolition (field units), civil engineering, water supply, and sewage (construction units), and in workshop and park activities. Boyle et al. [9] indicated that higher combat-zone engineer mortality was not seen in the US Vietnam Experience Study, but the number of engineers in that study was small (data were not shown in the article).

Two studies of female US Vietnam veterans (using the same cohort with different follow-up times) were available [23,51]. It is estimated that 5,000 to 7,000 US service women served in Vietnam, the majority of whom were nurses [23]. When compared with their respective controls, motor vehicle-related mortality appears to be higher among female Vietnam veterans than among male Vietnam veterans. This is despite the fact that the mortality rate ratios from all external causes were only slightly higher in the women.

### Injury-related mortality among First Persian Gulf War veterans

Table 4 summarizes the five retrospective cohort studies that examined the post-conflict injury-related mortality of First Persian Gulf War veterans. Because of the relatively short follow-up times (compared with the Vietnam studies) almost all investigations probably involved both discharged service members and those still active in service. Data provided in the study by MacFarlane et al. [32] indicate that, in their 8-year follow-up period, 34% of the cohort was likely still on active duty.

**Table 4:** Studies examining injury-related post-Persian Gulf service mortality (all investigations involve retrospective cohort designs)

	Study Characteristics				Mortality Rate Ratios Gulf Veterans/Non-Gulf Veterans (95% Confidence Intervals)			
	
Study [Reference No]	Follow-Up Period in Years^a^	Sample^b^	Sample Size^c^	Measure^d^	All External Causes (Injury)	Motor Vehicle Accidents	Suicide	Homicide
Kang and Bullman 1996 [28]	M = 3	♂ US Service Members	GV = 544,270 NGV = 456,726	AMRR^e^	1.17 (1.07–1.29)	1.27 (1.09–1.48)	0.88 (0.72–1.08)	0.80 (0.61–1.05)
				
		♀ US Service Members	GV = 49,919 NGV = 84,517		1.78 (1.16–2.73)	1.81 (0.96–3.41)	1.47 (0.63–3.43)	2.66 (0.96–7.36)

Kang and Bullman 2001 [57]	M = 7 A = 7	♂ US Service Members	GV = 578,369^h^ NGV = 646,997^h^	AMRR^f^	1.04 (0.99–1.10)	1.19 (1.09–1.30)	0.92 (0.83–1.02)	0.90 (0.78–1.04)
				
		♀ US Service Members	GV = 43,533^h^ NGV = 99,25^h^		1.39 (1.08–1.80)	1.63 (1.09–2.45)	1.29 (0.78–2.31)	1.54 (0.86–2.76)

MacFarlane et al. 2000 [32]	M = 8	UK Service Members	GV = 53,416 NGV = 53,450	MRR	1.18 (0.98–1.42)^i^	1.25 (0.91–1.72)	0.98 (0.65–1.48)	0.75 (0.11–4.44)

MacFarlane et al. 2005 [59]	M = 13	UK Service Members	GV = 51,753 NGV = 50,808	AMRR^g^	1.19 (1.02–1.39)	1.44 (1.13–1.84)^j^	1.04 (0.80–1.36)	no data in article

Sim et al. 2003 [61]	M = 10	Australian Service Members	GV = 1,833 NGV = 2,847	AMRR^i^	1.1 (0.5–2.9)	few cases no data in article	few cases no data in article	few cases no data in article

^a^M = Maximal follow-up period (the first number is the year the last person entered the study to the end of the survey period; the second number is the year the first person entered the study to the end of the survey period). A = Average follow-up time (if reported).

^b^US = United States, UK = United Kingdom, ♂ = male, ♀ = female

^c^GV = Gulf War veterans; NGV = Not Gulf War veterans

^d^MRR = mortality rate ratio; AMRR = adjusted mortality rate ratio

^e^Adjusted for age, gender, race, branch of service, and component

^f^Adjusted for age, race, service branch, component, and marital status

^g^Adjusted for age

^h^Estimated from demographics in article.

^i^Adjusted for age, rank, and service type

^j^Called "transportation accidents" using ICD-10

Studies of veterans of the First Persian Gulf War have been performed for US, UK, and Australian service members. The two US studies [28,57] compared virtually all service members serving in the Gulf War with a stratified random sample of about half of all service members (active duty, National Guard, and Reserves) serving outside the Gulf theater during the war. Table 4 shows that there was an excess of injury-related mortality among Gulf War veterans compared with non-Gulf War veterans. Much of this excess mortality is accounted for by motor vehicle-related events. Among US men and the UK and Australian cohorts (the latter two cohorts mostly men), deaths due to suicides and homicides were generally somewhat lower for the Gulf War veterans compared with non-Gulf War veterans.

About 50,000 US service women served in the Persian Gulf conflict, making up 7% of the total US force. The two US studies that specifically examined service women [28,57] suggested that the post-Gulf War mortality of women differed from that of the men. Table 4 shows that, when compared with their male counterparts, female Gulf War veterans had higher adjusted mortality rate ratios for all external causes, for motor vehicle accidents, and for suicide and homicide [28,57].

### Changes in mortality rates over time

Several investigations found that the excess post-conflict injury-related mortality in conflict-zone veterans diminishes over time. In the Vietnam Experience Studies [9,29], Vietnam veterans showed considerably reduced excess motor vehicle-related mortality after 6 to 14 years of follow-up and virtually no excess mortality when examining 6 to 30 years of follow-up, as shown in Table 5. Kang and Bullman [57] examined US Persian Gulf War veterans and separated motor vehicle accident mortality into four 20-month periods (1.7 years), as shown in Table 5. Similar to the Vietnam cohort [9,29], the mortality rate ratio for motor vehicle events decreased progressively over the 6.7 years of follow-up. Finally, MacFarlane et al. [59] examined temporal changes in all external cause mortality rate ratios for UK Gulf War veterans and found a reduction after 7 to 13 years of follow-up, as shown in Table 5. One exception to these trends of decreasing rates was a Vietnam Marine cohort [25] that showed similar mortality rate ratios (Vietnam Marines/non-Vietnam Marines) in the first 5 years of follow-up compared with later follow-up (up to 24 years) for all injuries and motor vehicle-related events. The authors [25] did not report the exact mortality rate ratios in their article.

**Table 5:** Temporal changes in injury-related mortality rate ratios (95%CIs) in various studies

Conflict	Study	Follow-up Period (years)	All External Causes^b^	Motor Vehicle Events^b^	Suicide (E950–959)^b^	Homicide (E960–969)^b^
Vietnam	Boyle et al. 1987 & Boehmer et al. 2004 [9,29]	≤ 5	ND	1.93 (1.16–3.22)	1.72 (0.76–3.88)	1.52 (0.59–3.91)
		
		6–14	ND	1.16 (0.72–1.87)	0.64 (0.32–1.30)	0.78 (0.39–1.55)
		
		6–30	ND	1.02 (0.73–1.43)	0.93 (0.64–1.34)	0.80 (0.50–1.26)

Gulf	Kang & Bullman 2001 [57]	≤ 1.7	ND	1.32 (1.13–1.53)	ND	ND
		
		1.8–3.3	ND	1.21 (1.01–1.45)	ND	ND
		
		3.4–5.0	ND	1.17 (0.98–1.40)	ND	ND
		
		5.1–6.7	ND	1.00 (0.82–1.22)	ND	ND
	
	MacFarlane et al. 2005 [59]	≤ 6	1.13 (1.06–1.63)	ND	ND	ND
		
		7–13	1.05 (0.83–1.33)	ND	ND	ND

^a^Values are mortality rate ratios (conflict-zone veterans/non-conflict-zone veterans) with 95% confidence intervals.

^b^ND = No data reported in article

### Meta-analyses

Meta-analyses were performed only for the retrospective cohort studies for two reasons. First, the proportionate mortality investigations had considerably lower vital status sensitivity (as discussed above). Second, the proportionate mortality investigations provide only the number of deaths as proportion of the total deaths and denominator data were not available as with the retrospective cohort investigations. No meta-analysis methods were found that allowed combining proportionate mortality ratios with mortality rate ratios [33].

Table 6 shows the univariate mortality rate ratios obtained or calculated from the retrospective cohort studies and used for the meta-analysis. In most cases, cohort-specific univariate rate ratios and confidence intervals were available in the article; in other cases, person-time data and the number of mortality cases were presented and these were used to calculate the univariate mortality rate ratios and confidence intervals [23,25,27,29,32,42,46,51,61]. Two articles provided univariate rate ratios, but did not provide confidence intervals [28,57]. In these two studies, rate ratios and confidence intervals could be calculated from data in the article but, presumably because of rounding error, the rate ratios were slightly different from those reported in the paper.

**Table 6:** Univariate mortality rate ratios obtained or calculated from retrospective cohort studies

		Mortality Rate Ratios Conflict-Zone Veterans/Non-Conflict-Zone Veterans (95%Confidence Intervals)			
		
Conflict	Study	All External Causes	Motor Vehicle Events^a^	Suicide^a^	Homicide^a^
Vietnam	Fett et al. 1984, [42]	1.26 (1.03–1.54)	1.17 (0.90–1.52)	1.51 (0.98–2.33)	ND
	
	Boyle et al. 1987 [9]	1.25 (1.00–1.55)	1.48 (1.04–2.09)	0.98 (0.58–1.65)	0.99 (0.57–1.71)
	
	Boehmer et al. 2004 [29]	1.19 (1.01–1.39)	1.24 (0.94–1.64)	1.03 (0.74–1.44)	0.90 (0.60–1.36)
	
	Watanabe et al. 1995 [25]	1.20 (1.00–1.46)	1.04 (0.76–1.43)	1.15 (0.75–1.76)	ND
	
	Dalager and Kang 1997 [27]	0.84 (0.59–1.22)	ND	ND	ND
	
	Thomas et al. 1991 [23]	1.26 (0.77–2.07)	2.44 (0.83–7.14)	1.00 (0.41–2.41)	ND
	
	Cypel and Kang, 2008 [51]	1.28 (0.88–1.85)	2.24 (1.08–4.64)	0.95 (0.45–1.83)	ND

Gulf	Kang and Bullman 1996 [28] ♀	1.26 (1.15–1.38)	1.41 (1.21–1.64)	0.92 (0.75–1.11)	0.95 (0.73–1.24)
	
	Kang and Bullman 2001 [57] ♀	1.07 (1.02–1.13)	1.25 (1.14–1.36)	0.98 (0.88–1.08)	0.99 (0.86–1.13)
	
	Kang and Bullman 1996 [28] ♂	1.95 (1.28–2.96)	1.88 (1.01–3.50)	1.56 (0.69–3.53)	3.12 (1.15–8.43)
	
	Kang and Bullman 2001 [57] ♂	1.43 (1.11–1.85)	1.65 (1.10–2.48)	1.27 (0.77–2.09)	1.72 (0.98–3.05)
	
	Sim et al. 2003 [61]	1.04 (0.50–2.31)	0.52 (0.10–2.57)	0.93 (0.22–3.90)	ND
	
	MacFarlane et al. 2000 [32]	1.18 (0.98–1.41)	1.25 (0.92–1.72)	0.98 (0.65–1.48)	0.75 (0.11–4.44)
	
	MacFarlane et al. 2005 [59]	1.18 (1.01–1.38)	1.43 (1.12–1.82)	1.04 (0.80–1.35)	ND

^a^ND = No data reported in article

For the meta-analysis, studies were grouped by length of follow-up and conflict. "Length of follow-up" was a relative term since the follow-up periods in the Vietnam studies were much longer than those of the Gulf-conflict studies. Two studies [23,27] were not included in the Vietnam meta-analysis because the cohorts in these investigations were used in a subsequent study [51]. For the Gulf meta-analysis, one study [61] was excluded because it overlapped the long and short follow-up periods; there was little difference in the SMRRs when the data from this study [61] were added to either follow-up period, because the study had such a small number of subjects relative to the other investigations.

Table 7 shows the SMRR comparing conflict-zone service members with non-conflict zone service members by conflict and length of follow-up. Methodological quality scores were averaged for the studies involved in each calculation and descriptive statistics are shown. In both the Vietnam and Gulf conflict studies, mortality from all external causes and from motor vehicle events was higher among the conflict-zone service members. In both the Vietnam and Gulf studies, mortality risk among conflict-zone service members decreased in the longer-term follow-up periods for all external causes and motor vehicle events, but mortality remained higher than among the non-conflict zone service members. The SMRRs for motor vehicle mortality were higher in the Gulf studies (reflecting higher risk for Gulf War conflict-zone service members) compared with the Vietnam studies, possibly because of the shorter follow-up periods in the Gulf cohorts.

**Table 7:** Meta-analysis: Summary mortality rate ratios comparing conflict-zone service members to non-conflict-zone service members

Conflict	Follow-Up Period (Studies)	Mortality Grouping	Summary Mortality Rate Ratio Conflict-Zone Service Members/ Non-Conflict-Zone Service Members (95% Confidence Interval)	Methodological Quality Score (mean ± SD)
Vietnam	Shorter Follow-up, 9 to 18 Years [9,42]	All External Causes	1.26 (1.08–1.46)	94 ± 3
			
		Motor Vehicle Events	1.27 (1.03–1.57)	
			
		Suicide	1.26 (0.91–1.77)	
			
		Homicide	1.02 (0.89–1.17)	
	
	Longer Follow-up, 18 to 40 Years [25,29,51]	All External Causes	1.19 (1.11–1.28)	86 ± 6
			
		Motor Vehicle Events	1.21 (0.99–1.47)	
			
		Suicide	1.06 (0.83–1.36)	
			
		Homicide	0.90 (0.60–1.36)^a^	

Gulf	Shorter Follow-up, 3 to 8 Years [28,32]^b^	All External Causes	1.26 (1.16–1.37)	86 ± 4
			
		Motor Vehicle Events	1.39 (1.22–1.60)	
			
		Suicide	0.95 (0.80–1.14)	
			
		Homicide	0.95 (0.73–1.23)	
	
	Longer Follow-up, 7 to 13 Years [57,59]^b^	All External Causes	1.09 (1.04–1.14)	85 ± 3
			
		Motor Vehicle Events	1.29 (1.18–1.40)	
			
		Suicide	0.99 (0.85–1.15)	
			
		Homicide	1.02 (0.89–1.17)^c^	

^a ^Data available from only one study [29]

^b ^For the two Kang and Bullman studies [28,57], men and women were entered as separate cohorts in the meta-analysis

^c ^Data available from only one study [57]

Suicide risk was slightly elevated for the conflict-zone Vietnam service members in the shorter follow-up (due primarily to elevated risk in the Australian cohort [42]), but was similar to that for the non-Vietnam service members in the longer-term follow-up. In the Gulf War studies, there was little difference in suicide risk between the conflict and non-conflict-zone service members. Homicide risk differed little between the conflict zone and non-conflict-zone cohorts by conflict or length of follow-up, although the data were limited to one study in the longer-term follow-ups.

## Discussion

The studies reviewed here compared post-deployment injury-related mortality of service members serving in conflict zones with the mortality of those who did not serve in the conflict zones. Findings were relatively consistent across two conflicts (Vietnam and the Gulf War) and among service members from the US, UK, and Australia. The findings indicated that the post-conflict injury-related mortality was higher among those serving in conflict zones and that much of this elevated mortality was associated with motor vehicle events. The excess mortality in conflict-zone veterans tended to diminish over time. Mortality among female conflict-zone veterans was higher than that among their male counterparts when compared with their respective non-conflict zone controls. Our analyses generally agree with that of Boyle et al. [52] who reviewed short-term mortality in Vietnam veterans and Kang et al. [58] who reviewed mortality in first Gulf War veterans.

A number of hypotheses have been proposed to account for the excess injury-related mortality among conflict-zone veterans [2,28,32,60,79,80]. These have been well articulated by Bell et al. [79], who put forward a model that encompassed and expanded on many of these hypotheses. One hypothesis is that those who served in combat areas may have more post-traumatic stress disorders (PTSD) or depressive symptoms that may contribute to injury-related mortality [9,28,40,57,79]. Definitions of PTSD can vary widely, resulting in a variety of prevalence estimates [81-85], but when the same criteria are applied, prevalence values are similar [82] for those serving in combat theaters: studies applying more restrictive criteria put estimates at 3%, and investigations using more liberal criteria put estimates at 11 to 16% [82,84,85]. A one- to nine-year follow-up study [86] of Vietnam veterans who received residential treatment for PTSD compared the mortality in this population to the general US population and found that veterans with PTSD had significant elevations for deaths due to all "accidents" (standardized mortality ratio (SMR) = 5.7, 95%CI = 3.9–8.1), motor vehicle events (SMR = 4.4, 95%CI = 2.1–8.0), and suicide (SMR = 4.0, 95%CI = 1.8–7.4). However, individuals in residential treatment are likely the most serious cases: those with severe symptoms, critical life dysfunctions, and multiple comorbidities [86]. Other studies [80,84,87] have compared Vietnam veterans with and without PTSD. These investigations indicated that Vietnam veterans diagnosed with PTSD were two to four times more likely to die of injury (external causes) compared with veterans without posttraumatic stress disorders, regardless of whether or not they served in the conflict zone [84,87]. This suggested that the post-traumatic stress itself was the factor increasing mortality. Since the incidence of post-traumatic stress is higher in conflict-zone veterans than non-conflict zone veterans [84,87], these studies suggest that post-traumatic stress may be associated with some of the excess mortality in conflict-zone veterans.

Some Gulf War studies noted that post-traumatic stress symptoms were higher among women who have served in the conflict zone compared with their male counterparts [88-90], but other studies have found only marginal gender differences [91-93]. A longitudinal study suggested that female gender was associated with increasing symptoms over time [88]. A history of assault prior to combat exposure has been shown to increase the incidence of post-traumatic stress disorders [94-96] and women who are Gulf War veterans reported more precombat physical and sexual abuse than men [97].

Another hypothesis [40,98] advanced to account for the excess injury-related mortality in conflict-zone veterans is that the physical and/or psychological trauma associated with war experiences may result in coping behaviors such as substance abuse that might increase injury risk. Vietnam veterans with traumatic combat exposure had a higher risk of drug and alcohol abuse after discharge compared with those without direct combat experience [99,100]. Most Vietnam veteran drug users gradually achieved spontaneous remission over 9 to 14 years [16], and this may be associated with the risk reduction over time (Tables 5 and 7). On the other hand, the proportion of Gulf War veterans who consumed alcohol did not differ from that of non-Gulf War veterans [101], but combat exposure was not considered. There was evidence for Vietnam-era veterans that death certificates underestimated the involvement of alcohol in motor vehicle accidents by a factor of 7, although no comparison was made between those who served in Vietnam and those who did not [102]. There were no differences in the blood alcohol content of deployed vs. nondeployed Gulf War veterans who died in motor vehicle events [103]. However, *nondeployed *Gulf War veterans who had a previous substance abuse-related hospitalization were more likely to die in a motor vehicle event than deployed veterans, but this may reflect the lower likelihood that those with substance abuse problems would be deployed [104]. In the Vietnam Experience Study [47], data on alcohol were available in 62% of motor vehicle deaths, and these data indicated that drinking did not account for the excess deaths. The finding of an excess of drug-related deaths among Vietnam veterans in the Vietnam Experience Study [29] suggests the possibility that some drug-related coping behaviors may have been associated with mortality, but the number of drug-related deaths was small (10% of all mortality in the entire cohort). Studies comparing Vietnam and non-Vietnam veterans have suggested that demographic and wider social factors were considerably more important than Vietnam service in post-service drug use [15,105-107].

Another hypothesis is that the excess mortality in conflict-zone veterans may be associated with various exposures that result in ill-defined diseases and syndromes that might affect factors like decision making, balance, navigation, reaction time, and the like [79,108,109]. One case-control study [110] examined associations between fatal motor vehicle crashes and exposure to low dosages of nerve agents during munitions demolitions in the Gulf War. Cases were in-theater Gulf veterans who had died of a motor vehicle event and controls were in-theater Gulf veterans matched by gender and by being alive at the end of the calendar year in which the case died. This study found no difference between cases and controls in the likelihood of low-level exposure to these agents. A follow-up case-control study found no difference in the proportion of deployed service members notified of this munitions demolition between those involved in fatal vehicle crashes (cases) and those not involved in fatal vehicle crashes (controls) [111]. Finally, the post-war hospitalization experience was similar for those exposed to low-level agents and those not exposed [112]. A large number of research studies have examined health complaints among returning Gulf War veterans, but have failed to demonstrate a unique Gulf War illness [11,60]. However, this lack of specificity does not negate the possibility that greater injury mortality of deployed veterans may be the indirect consequence of some yet unexplained syndrome or disease process. Compared with non-Gulf War veterans, Gulf War veterans self-reported more functional impairment, more limited employment, and more symptomology [101].

Another hypothesis is that war experiences alter the perception of risk, resulting in more risk-taking behavior [2,28,57,59]. One study [58] examined data from 549 deployed Gulf War veterans and 398 nondeployed veterans from the same period who died during motor vehicle events. Gulf War veterans used seat belts less often, wore motorcycle helmets less often, and made crash-avoidance maneuvers less often, and were more often speeding. Although the data are limited to this single study, the pattern tends to support the contention that Gulf War veterans may perceive risk differently and may engage in more risk-taking behavior [60]. The reduction over time in the excess injury-related mortality of conflict-zone veterans (Tables 5 and 7) appears to be consistent with the hypothesis of altered risk perception, suggesting that risky behavior decreases as conflict-zone veterans "re-adapt" to civilian life.

Another hypothesis is that the processes of choosing individuals for deployment result in the selection of risk takers who might be more prone to death from trauma after leaving the military [2,9,79]. There is some weak evidence that Gulf War Veterans are pre-disposed to risk and engage in risky behaviors both pre- and post-deployment. Bell et al. [98] noted that Soldiers deployed to the First Gulf War were more likely to be risk takers, as measured by pre-war hazardous duty pay incentives. However, this effect was not large (risk ratio (deployed/not deployed) = 1.02, 95%CI = 1.02–1.03, secondary data analysis) and it is likely that those receiving hazardous duty pay would be in more deployable occupational specialties (infantry, combat engineers, armor, etc.). The same data [98] also suggested that a composite measure that included self-reports of drunk driving, speeding, and not wearing a seat belt was slightly higher in deployed Soldiers (risk ratio (deployed/not deployed) = 1.34, 95%CI = 0.83–2.16).

Finally, it is possible that deployed veterans may have less favorable outcomes for a particular injury because of war-related comorbidities [79]. If this were the case, it might be possible to see more hospitalizations or outpatient visits among deployed service members compared with those not deployed. One study found no difference in the post-Gulf War hospitalization experience of deployed versus nondeployed service members who were on active duty during the survey [113]. A follow-up study that included reservists, National Guard, and separated service members found some increased risk for injury hospitalizations in the Department of Defense hospitals and California statewide systems, but not in the VA hospitals [114]. Compared with nondeployed Gulf War veterans, more deployed Gulf War veterans were smokers [101] and former service members who smoked had higher mortality from external causes compared with nonsmoking former service members [115]. Thus, there is limited and conflicting data on whether or not war-related comorbidities influence injury-related mortality.

An interesting aspect of the excess mortality among conflict-zone veterans is that it tends to diminish over time. While no hypothesis have been advanced to account for this in the literature, it would seem that the hypotheses above related to time-dependent factors would be the most productive to explore. It may be that individuals with specific survival or health problems become mortality cases early in the postdeployment period, leaving as survivors those with lower risk.

## Conclusion

This review established that injury-related mortality is higher in veterans deployed to combat zones, especially mortality related to motor vehicle events. Although the reasons for this elevated mortality cannot be determined from the data analyzed here, several hypotheses can be derived from the literature. Among these hypotheses, those dealing with stress-related symptoms, altered perceptions of risk, and war comorbidities seem the most promising explanations at this point. The excess mortality of combat veterans may not be linked to a single problem but may rather be multivariate in nature with multiple contributing factors. No research has been performed to account for the differences in the male and female mortality rate ratios and more work should concentrate on this. Before interventions can be designed to reduce the excess of injury-related mortalities additional attention should be focused on determining the etiology of these injuries and further testing of the specific hypotheses discussed here.

## List of abbreviations

AMRR: adjusted mortality rate ratio; APMR: adjusted proportionate mortality ratio; DD: Department of Defense; DMDC: Defense Manpower Data Center; ICD: International Classification of Diseases (numeral after abbreviation is revision number); IRS: Internal Revenue Service; MA: Massachusetts; MI: Michigan; MRR: mortality rate ratio; NCHS: National Center for Health Statistics; NDI: national death index; NPRC: National Personnel Records Center; NVA: Vietnam-era veterans not serving in Vietnam; NY: New York; PMR: proportionate mortality ratio; SSA: Social Security Administration; UK: United Kingdom; US: United States; VA-BILRS: Veterans Administration-beneficiary identification and record locator subsystem; VN: Vietnam-era veterans serving in Vietnam; WI: Wisconsin.

## Competing interests

The authors declare that they have no competing interests.

## Authors' contributions

JJK conceived the project, drafted the manuscript, performed literature review, and made intellectual contributions to the paper. REM assisted with the literature review and made intellectual contributions to the writing of the paper. TLG assisted with the literature review, assisted with the statistical analysis, and made intellectual contributions to the writing of the paper. BHJ assisted with the literature review and made intellectual contributions to the writing of the paper. All authors have read and approved the final manuscript.

## Pre-publication history

The pre-publication history for this paper can be accessed here:


